# IMUs Can Estimate Hip and Knee Range of Motion during Walking Tasks but Are Not Sensitive to Changes in Load or Grade

**DOI:** 10.3390/s24051675

**Published:** 2024-03-05

**Authors:** AuraLea Fain, Ayden McCarthy, Bradley C. Nindl, Joel T. Fuller, Jodie A. Wills, Tim L. A. Doyle

**Affiliations:** 1 Biomechanics, Physical Performance and Exercise Research Group, Department of Health, Medicine and Human Sciences, Macquarie University’s Biomechanics, Sydney, NSW 2113, Australia; auralea.fain@hdr.mq.edu.au (A.F.); ayden.mccarthy@hdr.mq.edu.au (A.M.); joel.fuller@mq.edu.au (J.T.F.); jodie.wills@mq.edu.au (J.A.W.); 2 Neuromuscular Research Laboratory/Warrior Performance Center, Department of Sports Medicine, University of Pittsburgh, Pittsburgh, PA 15260, USA; bnindl@pitt.edu

**Keywords:** inertial measurement units, wearable sensors, kinematics, algorithm development

## Abstract

The ability to estimate lower-extremity mechanics in real-world scenarios may untether biomechanics research from a laboratory environment. This is particularly important for military populations where outdoor ruck marches over variable terrain and the addition of external load are cited as leading causes of musculoskeletal injury As such, this study aimed to examine (1) the validity of a minimal IMU sensor system for quantifying lower-extremity kinematics during treadmill walking and running compared with optical motion capture (OMC) and (2) the sensitivity of this IMU system to kinematic changes induced by load, grade, or a combination of the two. The IMU system was able to estimate hip and knee range of motion (ROM) with moderate accuracy during walking but not running. However, SPM analyses revealed IMU and OMC kinematic waveforms were significantly different at most gait phases. The IMU system was capable of detecting kinematic differences in knee kinematic waveforms that occur with added load but was not sensitive to changes in grade that influence lower-extremity kinematics when measured with OMC. While IMUs may be able to identify hip and knee ROM during gait, they are not suitable for replicating lab-level kinematic waveforms.

## 1. Introduction

Quantifying movement patterns outside the confines of a controlled laboratory environment is highly important in the field of biomechanics. Advancements in hardware analytics have increased the viability of using wearable sensors, such as inertial measurement units (IMUs), to quantify meaningful metrics associated with mechanical load (e.g., surrogate measures of ground reaction forces) and kinematics outside of a laboratory [1,2,3,4,5,6]. These advancements have made sensors more discrete and suitable for real-world scenarios than earlier models and have expanded the data that can be derived from sensors in both quality and quantity. While it is generally accepted that 3D-marker-based optical motion capture (OMC) is the industry gold standard for the measurement of movement kinematics, this method restricts studies to a laboratory space, which rarely provides an ecologically valid environment [7]. Though controlled studies are necessary to understand the mechanics of human movement in isolation, measuring movement strategies in an organic environment is best suited to accurately representing motions generated in scenarios where injuries occur compared with laboratory settings. The gap between the laboratory and the real world has led to many researchers endeavouring to employ IMUs to measure human movement patterns.

Despite the promise of applying IMUs in field-based settings, relatively few studies have used them to investigate joint motion. A recent scoping review found that, of over 200 studies employing IMUs for running gait analysis, only 10% investigated joint angles or joint range of motion [8]. To explore single joint kinematics, two sensors, one placed proximally and the other distally to the joint of interest, can quantify clinically relevant measures [9,10]. Alternatively, whole-body kinematics while executing athletic and tactical-specific tasks can be measured by employing a full-body suit with embedded IMUs [2,11,12]. However, whole-body kinematics measured in this way may be impractical, as a suit is cumbersome in real-world scenarios, and many injuries in both athletic and tactical populations occur in the lower extremities, highlighting the practical relevance of focusing specifically on the lower limbs [13,14,15]. As such, it is evident that there is a dearth of literature exploring an accessible sensor fusion approach, particularly with market-available sensors, to quantify joint-level kinematics. In a controlled laboratory environment, it is reported that such an IMU methodology can, with moderate accuracy, measure lower-body (hip, knee, and ankle joint) kinematics compared with OMC, particularly during discrete movements, including squatting tasks and countermovement jumps [1,3,12]. However, it is unclear if this validity carries over to continuous locomotion tasks that have high physiological demands and continue for extended time periods, particularly for military personnel.

A leading cause of military injury is long-duration marches with the addition of external load, a task that also necessitates navigation over variable terrain [13,15]. A recent review cites that nearly 80% of all injuries incurred during basic training occur in the lower extremities and may be associated with biomechanical maladaptation to account for the addition of load during walking tasks [15]. Studies have explored the influence of load and grade during locomotor tasks that are common in military environments, finding significant differences between load and terrain conditions [16,17,18]. However, most of these studies use OMC, which necessitates investigation in a sterile lab environment. Being able to utilize IMUs to measure movement patterns outside of a lab environment may be particularly advantageous to military personnel who complete these tasks outdoors. Few studies have explored the use of IMUs to quantify metrics that may be associated with injury in military personnel. Accelerations (vertical and resultant) derived from a single commercially available IMU placed on the distal tibia had a moderate relationship with ground reaction force metrics during a simulated loaded march [5]. Alternatively, an entire IMU suit has been trialled to quantify whole-body kinematics during military-specific tasks, including running and walking [2]. While these studies examined validity with load, it is unclear if there are load-induced changes in IMU signals, similar to those seen in OMC-based metrics [18]. Furthermore, both studies employed only flat ground for the locomotor task. As such, it is unclear if these IMU systems are sensitive to kinematic changes that occur because of load or grade changes.

While Fain et al. [4] showed that a minimal sensor approach originally developed by Hindle et al. [1] can quantify movement patterns in discrete tasks, it is unclear if this IMU methodology transfers to continuous gait tasks that mimic scenarios specific to military personnel. IMUs need to be sensitive to changes in the mode of gait, load, and grade to provide useful quantification of common military tasks. As such, the aims of this study were two-fold to explore (1) the validity of a seven-sensor IMU system to quantify lower extremity kinematics during walking and running and (2) if this IMU system has similar sensitivity to OMC in detecting kinematic changes that may occur as a consequence of grade, load, or a combination of the two.

## 2. Methods

### 2.1. Participants

Twenty participants volunteered to participate in this study. One participant dropped out because of an injury between enrolment and collection. As such, data from 19 individuals are presented (26.3 ± 6.7 years, 1.70 ± 0.09 m, 71.3 ± 15.4 kg, 11 male, 9 female). All participants were self-reportedly free from current injuries and able to safely don 23 kg in the form of a weighted vest. All participants provided written informed consent prior to data collection. This study received institutional approval (HREC Project Number: 52022787737978).

### 2.2. Procedures

Each data collection session included gait at two different speeds (walking: 4.5 kph; running: 8.1 kph), and three different grades (flat: 1%, uphill: +6%, downhill −6%) on a treadmill instrumented with two tandem 6-axis force platforms (AMTI, Watertown, MA, USA). A grade of 1% was chosen for “flat” to mimic the demands of outdoor locomotion [19]. For event identification, heel strike and toe-off were defined as the instant the instrumented treadmill force exceeded and fell below 20 N, respectively. The order of speed and grade was standardized for each participant, while the load condition was randomized. The order grades were completed in was flat, uphill, and then downhill. Walking was always completed first, followed by running, before moving to the next grade. As such, testing was: flat—walking, running; uphill—walking, running; downhill—walking, running. If the participant was completing the loaded condition, they donned a 23 kg weighted vest.

#### 2.2.1. OMC

**Figure 1:**
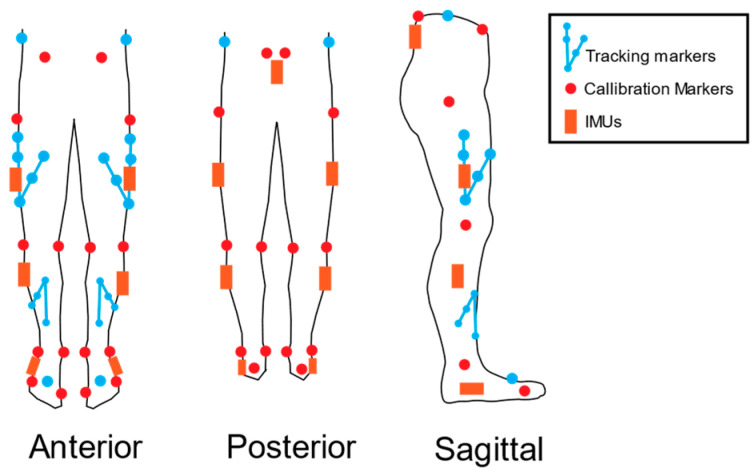
Placement of OMC retroreflective markers and IMUs on each participant.

Trajectories of 40 retro-reflective markers and clusters placed on specific anatomical locations were tracked with 10 high-speed optical infrared cameras (MX-T40 S, 250 Hz, Nexus v.2.12.1, Vicon Motion Systems Ltd., Oxford, UK) (Figure 1) [19]. From the static trial, a participant-based kinematic model was generated with bilateral feet, shank, thigh, and pelvis with a joint coordinate system located at each joint centre (Visual 3D, C-Motion, v.2021.11.3). Functional hip and knee joint centres were calculated according to Schwartz and Rozumalski following static calibration, while the midpoint between the medial and lateral malleoli during the static calibration was used to define the ankle joint centre according to Wu et al. [20,21]. All joint angles were defined as rotations of the distal segment in relation to the proximal segment. Once functional trials were completed, calibration markers were removed, and only tracking markers remained on each participant. All OMC data were filtered with a lowpass 4th-order Butterworth filter at 12 Hz, and gaps in the trajectory data were filled in Vicon Nexus (v.2.12.1, Vicon Ltd., London, UK) using spline and rigid body filters.

#### 2.2.2. IMUs

Bluetooth connection between the IMUs and the data collection computer enabled IMU data to be collected synchronously with both OMC and force plate data in Vicon Nexus (iMeasureU, Vicon Ltd., London, UK). All IMUs were placed according to Hindle et al. [2] (Table 1; Figure 1). The IMUs had 9 degrees of freedom, collecting data from an onboard triaxial accelerometer (±16 g, 1125 Hz), magnetometer (±4900 μT, 112.5 Hz), and gyroscope (±2000°/s, 1125 Hz). To calibrate the magnetometers, IMUs were independently removed and rotated around each axis at the end of the data collection session to generate a magnetic calibration file to be used for analysis.

IMU data were exported from Vicon in a CSV file that up-sampled data to 1250 Hz to maintain time synchronization with the OMC system collecting optical and force plate data at 250 and 1250 Hz, respectively. To derive joint kinematics, raw CSV data were loaded into a custom MatLab application that utilized data from all sensors embedded within the IMU (Appendix A). This MatLab application uses the same sensor fusion methodology originally developed by Hindle et al. and validated by Fain et al. [1,4] but was customized to enable users to manipulate data processing parameters. Within the application, the user is prompted to define a region of interest (by either time or frame) and filtering parameters. For analysis, the initial 10 s of data were omitted, and only data from the middle 20–30 s of the trial were used. A 4th-order Butterworth filter with a cut-off frequency of 6 Hz was used to filter IMU data. While human movement rarely exceeds 12 Hz when markers and IMUs are placed on bony landmarks (e.g., the tibia), the given IMUs were placed in locations much more susceptible to soft tissue wobble. As such, internal analysis determined that a 6 Hz cut-off frequency for the IMU kinematic waveforms was suitable. Instances of heel strike to define each stride were indexed from the outputs from Visual 3D. A custom MatLab code was used to identify peak flexion and extension angles and joint range of motion (ROM) in the sagittal plane for each joint, stride, and participant during the 10 s of interest. Only right-side data were analysed for both OMC and IMU data, as it is reported that there are no kinematic differences between limbs during gait [22].

### 2.3. Statistical Analyses

To compare waveforms of sagittal plane joint kinematics, one-dimensional Statistical Parametric Mapping was completed using open-source MatLab code from spm1D (Version M.0.4.10, spm1d.org) [23]. To enable comparison between OMC and IMUs reporting data at two different sampling frequencies (250 and 1250 Hz, respectively), filtered kinematic data were time-normalized to 101 points using linear time interpolation for each stride (toe-off to toe-off) and averaged across the strides to generate a participant-based mean kinematic waveform for each condition [24]. Using the spm1D method, between-system validity was measured by a series of paired-sample t-tests for each condition (e.g., OMC vs. IMU for flat, unloaded walking) that compared the averaged kinematic waveform for each system. To test if OMC and IMU kinematic waveforms were similarly sensitive to changes in load and grade, a spm1D 2-way repeated measures ANOVA was used separately for OMC and IMU data to compare the main and interaction effects of load (unloaded, loaded) and grade (flat, uphill, downhill). When reporting SPM analyses, all results are reported as a percentage portion of stride wherein there was a significant divergence of signals (i.e., they differed between 10 and 15% of stride).

Three separate repeated measures (RM) ANOVAs were completed for both walking and running to test for the main and interaction effects of the data source (*IMU*, *OMC*), load (*unloaded*, *loaded*) and grade (*flat*, *uphill*, *downhill)* on joint peak angles and ROM at the ankle, knee, and hip. The influence of load and grade was also investigated for each data source, independently. Where significant differences were observed, simple pairwise comparisons with Bonferroni correction were implemented. All RM ANOVA analyses were conducted using SPSS (IBM, v29.0.0). For comparisons, validity was determined as a statistically insignificant difference between the IMU and OMC waveforms and discrete variables (peak angles and ROM). Sensitivity was determined by the ability of the IMU system to replicate grade- and load-induced changes to waveforms and discrete variables. Root mean square error (RMSE) was also employed to explore the magnitude of system-based differences in discrete variables.

## 3. Results

### 3.1. Validity

**Figure 2:**
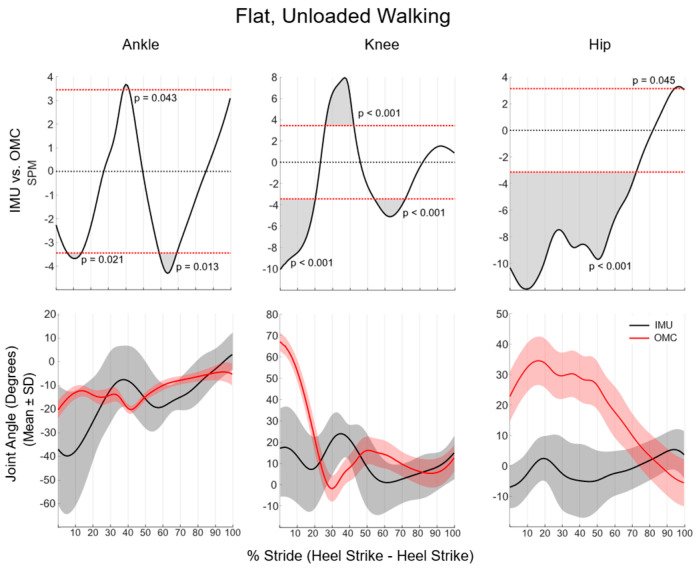
SPM analysis comparing IMU and OMC during walking. Shaded portions above and below the horizontal red lines (*p* < 0.05) (**top row**) indicate significant differences between the IMU and OMC mean kinematic waveforms (**bottom row**).

During flat, unloaded walking, SPM analyses of the sagittal plane revealed that ankle kinematics were significantly different between systems for 23% of the total stride (from 4 to 13%, 39 to 41%, and 60 to 72%), knee kinematics were different for 57% of the stride (from 0 to 20%, 23 to 41%, and 55 to 74%), and hip kinematics were different for 78% of the stride (from 0 to 75% and 97 to 100%) (Figure 2). Sagittal plane lower-extremity kinematic waveform differences between systems were not statistically significant from midstance to toe-off (75–100% of stride) for all lower extremity joints.

**Figure 3:**
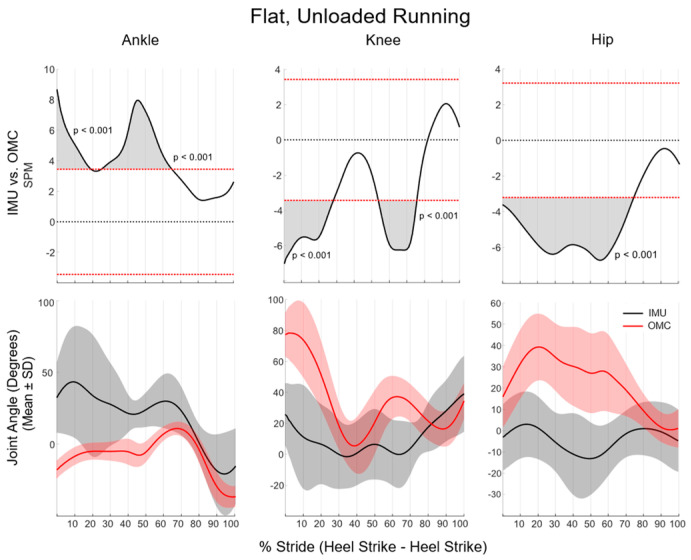
SPM analysis comparing IMU and OMC during running. Shaded portions above and below the horizontal red lines (*p* < 0.05) (**top row**) indicate significant differences between the IMU and OMC mean kinematic waveforms (**bottom row**).

SPM analyses during flat, unloaded running revealed that sagittal plane ankle kinematics were significantly different between systems for 67% of the running stride (from 0 to 20% and 28 to 75%). Knee kinematics were significantly different between systems for 50% of the running stride (from 0 to 27% and 57 to 80%), while hip kinematics significantly differed between IMU and OMC for 80% of the running stride (from 0 to 80%). As in walking, sagittal plane lower-extremity kinematic waveform differences between systems were not statistically significant from midstance to toe-off (75–100% of stride, Figure 3).

During walking, paired-sample *t*-tests revealed significant differences between IMU and OMC for all discrete joint kinematics except for knee and hip ROM (Table 2). IMUs overestimated all ankle kinematics, as well as knee and hip extension, but underestimated knee and hip flexion.

While running, IMUs significantly overestimated all discrete joint kinematics when compared with OMC except for peak hip flexion (Table 3). RMSE for peak hip flexion was over 28 degrees and implied that the systems were not consistently comparable for this outcome. The lowest RMSE was observed for peak knee flexion, though systems were significantly different (*p* = 0.040). All other RMSE values were greater than 21 degrees, with the highest RMSE observed for ankle ROM with a value of nearly 80 degrees.

### 3.2. Sensitivity

Graphs and tables that demonstrate system sensitivity can be found in the supplemental material (Tables S1a–S2b and Figures S1–S6). Grade significantly influenced IMU knee kinematic waveforms between 25 and 37% of walking stride, with downhill having greater knee flexion than flat (*p* = 0.006; Figure S2). Neither load nor grade significantly altered any other IMU kinematic waveforms during walking. Load influenced OMC kinematic waveforms during walking for the following joints: load reduced ankle sagittal plane motion at the ankle from 21 to 35% of the stride (*p* < 0.001) (Figure S1); load influenced sagittal plane kinematics at the knee for 72% of the stride (1–36%, 38–72%, and 98–100%; *p* ≤ 0.043) (Figure S2); and load increased sagittal plane motion at the hip for 79% of the stride (2–81%; *p* < 0.001) (Figure S3). Grade influenced OMC kinematic waveforms during walking for the following joints: the ankle in flat compared with uphill for 79% of the stride (16–95%; *p* < 0.001), in flat compared with downhill for 33% of the stride (1–6%, 42–63%, 82–87%, and 99–100%; *p* ≤ 0.5) (Figure S1); the knee in flat compared with uphill for 61% of the stride (1–4%, 20–71%, and 94–100%; *p* < 0.001) and in flat compared with downhill for 7.4% of the stride (1–21%, 35–37%, and 48–100%; *p* ≤ 0.048) (Figure S2); and the hip in flat compared with uphill for 78% of the stride (2–80%; *p* < 0.001) and flat compared with downhill for 40% of stride (7–47%; *p* < 0.001) (Figure S3).

While running, load significantly increased sagittal plane motion in ankle IMU kinematic waveforms for 26% of the stride (68–94%; *p* < 0.001) (Figure S4). Load did not influence IMU kinematic waveforms at the knee or hip (Figures S5 and S6). Grade influenced IMU hip kinematic waveforms but only increased hip flexion from 21 to 27% of the running stride in flat compared with downhill (Figure S6). Grade did not influence any ankle or knee kinematic waveforms as quantified by IMUs. Conversely, OMC demonstrated significant differences between load and grade for each joint. Load influenced OMC kinematic waveforms during running for the following joints: load increased sagittal plane motion at the ankle for 59% of the stride (16 to 35%, 54 to 66%, and 72 to 100%; *p* ≤ 0.007) (Figure S4); load increased sagittal plane motion at the knee for 12% of the stride (79–91%; *p* = 0.019); and load increased sagittal plane motion at the hip for 84% of the stride (7–91%; *p* < 0.001) (Figure S6). Grade influenced OMC kinematic waveforms during running for the following joints: ankle kinematics were influenced in flat compared with uphill for 54% of the stride (28–39% and 41–84%; *p* ≤ 0.004) and downhill for 43% of the stride (1–3%, 44–77%, 93–100%; *p* ≤ 0.042) (Figure S4); knee motion was influenced by grade in flat compared with downhill from 84 to 100% of the stride (*p* = 0.003) (Figure S5); and hip motion was influenced by grade in flat compared with uphill from 1 to 67% of the stride (*p* < 0.001) and downhill from 4 to 24% of the stride (*p* < 0.001) (Figure S6).

#### 3.2.1. IMU

The influence of load and grade on IMU kinematics for walking and running can be found in Supplemental Tables S1a and S1b respectively.

Grade significantly influenced IMU ankle dorsiflexion (*p* = 0.019) and ROM (*p* = 0.013), with flat having less dorsiflexion (*p* = 0.002) and ROM (*p* = 0.009) than downhill. Grade influenced IMU knee flexion (*p* = 0.001) and ROM (*p* < 0.001). Uphill had greater knee flexion than downhill (*p* = 0.008), and downhill had greater ROM than both flat (*p* = 0.030) and uphill (*p* = 0.004). Load increased knee ROM only (*p* = 0.012).

While running, grade significantly influenced IMU knee extension (*p* = 0.006), with greater extension in uphill compared with downhill (*p* = 0.029). While a significant effect of grade was observed on IMU knee ROM, post hoc analyses revealed no significant differences. Hip extension (*p* < 0.001) and ROM (*p* = 0.004) were greater in unloaded compared with loaded.

#### 3.2.2. OMC

The influence of load and grade on OMC kinematics for walking and running can be found in Supplemental Tables S2a and S2b respectively.

While walking, there was a significant main effect of grade on OMC ankle plantarflexion (*p* = 0.002), dorsiflexion (*p* < 0.001), and ROM (*p* < 0.001) during walking. OMC plantarflexion was greater in uphill compared with flat (*p* < 0.001), and OMC dorsiflexion and ROM were greater in uphill compared with flat (*p* = 0.016 and *p* < 0.001, respectively) and downhill (*p* < 0.001 and *p* < 0.001, respectively) and greater in flat compared with downhill (*p* < 0.001 and *p* = 0.002, respectively).

There was a significant effect of grade on running OMC ankle plantarflexion, dorsiflexion, and ROM (*p* < 0.001). Uphill had greater plantarflexion and ROM than flat and downhill, and flat had greater plantarflexion and ROM than downhill (all *p* < 0.001). For running OMC dorsiflexion, downhill had greater dorsiflexion than both flat and downhill (*p* < 0.001 and *p* = 0.006, respectively).

There was an interaction effect of load and grade on OMC knee extension (*p* = 0.044) and ROM (*p* = 0.002). Unloaded uphill walking had less knee extension than both flat and downhill (*p* < 0.001 and *p* = 0.044, respectively). While in the loaded condition, only flat had greater knee extension than uphill (*p* = 0.044). For all grades, unloaded had greater knee extension than loaded (*p* ≤ 0.003). In the unloaded condition, downhill had greater ROM than both uphill and flat (*p* < 0.001), and flat had greater ROM than uphill (*p* < 0.001). Unloaded had greater ROM than loaded only in the flat condition (*p* = 0.028). Knee extension and flexion were greater in the unloaded condition compared with the loaded condition (both *p* < 0.001). A main effect of grade was observed for extension (*p* < 0.001), flexion (*p* < 0.001), and ROM (*p* < 0.001). Flat and downhill had greater knee extension than uphill (both *p* < 0.001), there was less knee flexion in downhill compared with both flat and uphill (both *p* < 0.001), and uphill had significantly less ROM than both flat and downhill (both *p* < 0.001), while flat had significantly less ROM than downhill (*p* = 0.001).

In running, there was an interaction effect of load and grade on OMC knee ROM (*p* = 0.015), with ROM significantly higher in uphill compared with flat in the unloaded condition only (*p* = 0.036) and lower in the flat condition compared with the downhill condition with the addition of load (*p* = 0.020). Unloaded OMC knee ROM was significantly higher in the unloaded condition compared with the loaded condition in flat (*p* = 0.018) and uphill (*p* = 0.002) but not downhill. Knee extension and flexion were higher in flat compared with uphill (*p* = 0.025 and *p* < 0.001, respectively), while knee ROM was greater in uphill compared with flat (*p* = 0.004). Knee extension was greater in downhill compared with both flat and uphill (both *p* < 0.001), while knee flexion was significantly greater in downhill compared with uphill (*p* < 0.0050). For both knee extension and ROM, the unloaded condition was greater than the loaded condition (*p* < 0.001 and *p* = 0.008, respectively).

In walking, there was an interaction effect of grade and load on hip OMC ROM (*p* = 0.040). In both the unloaded and loaded conditions, uphill had greater ROM than both flat and downhill, while flat had greater ROM than downhill (all *p* < 0.001). Unloaded ROM was less than ROM with the addition of load for flat (*p* = 0.028), uphill (*p* < 0.001), and downhill (*p* = 0.001). There was a main effect of grade on hip flexion and ROM (both *p* < 0.001), with uphill having greater flexion and ROM than flat and downhill, while flat had greater flexion and ROM than downhill (all *p* < 0.001).

While running, an interaction effect of grade and load was observed for OMC hip flexion (*p* < 0.001) and ROM (*p* = 0.005). As in walking, running uphill had greater OMC flexion and ROM than flat and downhill, and flat had greater flexion and ROM than downhill (all *p* < 0.001). OMC hip flexion and ROM were significantly higher in the loaded condition compared with the unloaded condition for flat (both *p* < 0.001), uphill (*p* < 0.001), and downhill (*p* < 0.001 and *p* = 0.003, respectively). Uphill running hip flexion and ROM were significantly higher than flat and downhill and flat compared with downhill (all *p* < 0.001). Hip flexion (*p* = 0.008), extension (*p* < 0.001), and ROM (*p* < 0.001) were all higher in the loaded condition compared with the unloaded condition.

## 4. Discussion

Though this IMU system has previously been validated for generating valid kinematics compared with OMC for discrete tasks, similar validity was not observed in the walking or running tasks of the current study [1,4]. Discrete knee and hip ROM measures demonstrated similarity between systems for walking via the mean differences observed. However, large RMSE values and subsequent waveform analyses may suggest the IMU sensor system does not consistently replicate OMC for the entirety of the stride, particularly at the ankle and during running. Additionally, the proposed IMU system was not sensitive to kinematic changes caused by load or grade in the same manner as OMC in either gait mode.

IMUs overestimated both dorsiflexion and plantarflexion in the ankle while walking and running, resulting in significant system differences in ROM as quantified by discrete variables. Similar levels of poor agreement in ankle kinematics between IMU and OMC systems have been reported in dynamic tasks, including change of direction and jump-landing [3,12]. The differences between systems are not surprising given the high accelerations experienced by the bones of the distal limb during both walking and running. It is reported that nearly 65% of studies that used IMUs for gait analyses reported the axial acceleration magnitude of sensors placed on either the foot or the anterior aspect of the tibia [8]. While this use of IMUs is a robust measure of impact acceleration experienced by those running, this distal limb placement is subject to the high-magnitude accelerations experienced by the bony aspect of the limb during initial contact [25,26]. As such, it stands to reason that an algorithm that employs accelerometry to estimate kinematics results in a significant alteration of kinematics in tasks with high-impact-induced accelerations. While they are not the only factor, high accelerations may have influenced the large magnitude of standard deviations in the kinematic waveforms at the ankle, as visually represented in Figure 1 and Figure 2. While OMC standard deviations for plantarflexion, dorsiflexion, and ROM at the ankle during flat, unloaded running were 8.5°, standard deviations were as high as 33.6° for IMU metrics (Table 2). A lack of statistically significant differences in ankle kinematic waveforms during flat, unloaded running may also stem from large variations in the IMU-derived kinematic data. Specifically, ankle kinematic waveforms between systems were only significantly different at three small portions of the walking stride waveform (a cumulative 20% of stride) and from toe-off to roughly midstance (75% of stride) while running. Specifically, in walking, the standard deviation band of IMU data ranges from 15° to 60° compared with 4° to 10° of the OMC standard deviation band. These standard deviations in running are even larger, ranging from 20° to 75°. Effectively, SPM analysis identifies where the standard deviations of each data source no longer overlap. As such, the large variability in the data source (as observed with IMU kinematics) may be the underlying factor in a lack of statistically significant differences in IMUs compared with OMC.

The current study found that differences between IMU and OMC kinematic waveforms generally dissipated during midstance, aligning with the findings reported by Hindle and colleagues. Though the authors reported good system agreement in discrete tasks, dynamic tasks (e.g., bear crawls) significantly differed between IMUs and OMC outside of “stance” [1]. However, Hindle reported greater agreement with peak angles and ROM compared with the current study during continuous tasks, such as a shuffle walk and bear crawl [1]. There are several factors that may explain the divergence in the current study compared with Hindle et al. The two continuous tasks completed by Hindle et al. (i.e., the shuffle-walk and bear crawl) likely result in much lower impact accelerations compared with overground walking or running, especially downhill and/or with the addition of load. Running results in axial and resultant accelerations two and nearly four times higher than that of walking, respectively, and downhill running impact peaks are often more severe and taller than active peaks during gait on even or uphill terrain [16,25,26]. Improved agreement in discrete tasks and waveforms in walking compared with running supports this notion. As such, it may be that, at present, this IMU methodology is best suited for low-impact tasks and/or stationary tasks (e.g., walking and squats).

Though the IMUs over-estimated knee and hip extension, the underestimation of knee and hip flexion resulted in ROMs that were not significantly different from OMC. However, this did not continue during running, as flexion, extension, and ROM between systems were significantly different at both the knee and the hip. Just as the initial shock of impact forces elevates the magnitude of accelerations at the foot, the signal at the shank and thigh may have been significantly impacted by soft tissue movement artefacts. Such movement artefacts have long been an issue in motion capture, with large proximal tissues of the leg resulting in up to 5.4 cm of movement during a dynamic task [27]. This notion is supported by LaFortune and colleagues [28], who reported that accelerometers mounted on skin experienced accelerations two times greater than bone-mounted accelerometers. This comparison looked at placement on the bony aspect of the tibia, and IMUs of the current study were placed on the lateral midline of the shank and thigh—locations that may exhibit significant soft tissue movement artefacts at initial contact.

The secondary aim of this study endeavoured to explore the IMU system’s ability to identify kinematic differences that occur because of load and grade. There was an observed inconsistency and divergence between systems for measures significantly altered by load and/or grade conditions. With OMC, ankle dorsiflexion and ROM and hip ROM were significantly different between all grades while walking, while knee extension and hip flexion and ROM were significantly different between grades while running. Conversely, IMUs only detected differences in ankle dorsiflexion and ROM in flat compared with downhill walking, and there was no influence of grade on IMU-based running kinematics. A lack of differences between grades for the IMU kinematics during running may lie in the high accelerations experienced with the initial foot contact while running, increasing variability and decreasing the validity of IMU kinematic measures. Just as there were inconsistencies related to the influence of grade on kinematics, the influence of load was not parallel between systems. Load significantly influenced OMC knee extension while walking and hip flexion and ROM while running. While IMUs also detected load-based differences in hip ROM during running, they also detected differences in knee ROM while walking and ankle plantarflexion, which were not detected by OMC and were unlikely to represent real differences. Though SPM analysis revealed significant influences of load and grade on all OMC joint kinematic waveforms in both walking and running, similar patterns were not replicated in IMU kinematic waveforms; only grade significantly influenced knee kinematics for approximately 15% of the walking stride (Figure S2), while load and grade influenced running ankle kinematics for 30% and 9% of the running stride, respectively (Figure S1). The inability of this system to detect grade- and load-based differences may, again, stem from large standard deviations in the data set, which would make the precise detection of real differences challenging.

The large standard deviations observed at all joints might stem from variables that are less likely to be controlled with IMUs compared with OMC. While OMC has derived ways to minimize the effects of soft tissue artefacts (e.g., calibration markers vs. tracking markers), a similar standardized approach is yet to be accomplished by IMUs. Further, it is unclear how sensitive this system is to small placement variations between individuals and sessions. While placement was standardized, anatomical differences—particularly the lateral thigh musculature and/or subcutaneous fat—may lead to larger discrepancies in kinematic measures between individuals than kinematics quantified by markers placed on bony locations. Previously validated IMU systems, such as a commercially available IMU suit, can generate valid kinematics during military-specific tasks and dynamic tasks [2,3,11,12]. However, as reported by Nijmeijer et al., the standard deviations of the amplitude differences range between 45 and 60 degrees for the sagittal hip, knee, and ankle angle [13]. While the means of these systems are somewhat close, it is apparent that the IMU technology has a wide margin of error when it comes to quantifying kinematics as valid compared with those captured by OMC.

Deviations between IMU and OMC joint angle values may stem from distortions of the magnetic field within the lab throughout the data collection session. The custom-generated Matlab application employs data from the sensors’ magnetometers for orientation on the individual’s segment. While all data collection sessions are completed in the same laboratory environment, it has been reported that laboratories dedicated to biomechanics research are susceptible to changes in the magnetic field due to the large number of electronic devices that are employed in this type of research [29,30]. For example, data collections lasting longer than 20 s may experience significant magnetic distortion, and the standard deviation of the sensor orientation may be nearly 30 degrees at 5 cm above the floor level compared with 3 degrees when the sensor is at or above 100 cm above floor level [30]. While it has been reported that magnetic fields do not significantly alter sagittal plane joint kinematics when comparing indoor to outdoor walking tasks, this study was only conducted on one individual and merits larger-scale exploration [30].

Lastly, while enabling the authors to compare waveforms, SPM analysis is not without its limitations. Put simply, SPM analysis highlights where standard deviations being compared deviate from each other. The two different data sets presented drastically different standard deviations throughout the gait cycle, with a much larger spread of data with IMU-based kinematics compared with OMC-based kinematics. This highlights that caution must be taken when interpreting SPM findings associated with validity. While IMUs may be capable of reporting valid ranges of motion, they necessitate refinement to mimic waveform patterns, as well as minima and maxima.

## 5. Conclusions

This seven-sensor IMU system may estimate hip and knee range of motion with modest precision during walking. However, analyses of running suggest that the IMU system fails to generate kinematic measures that agree with those captured by a lab-based OMC system. Further, IMUs are not as sensitive to OMC-quantified altered kinematics that occur because of changes in load and grade. It may be that the technology and/or analysis software needs further development, particularly to account for movement artefacts associated with soft tissue perturbations and/or distortions of the IMU magnetic field. It is possible that IMUs may not be a suitable technology to alone replicate lab-based kinematics; instead, there may be other metrics or patterns in the IMU signals that warrant further investigation.

## Figures and Tables

**Table 1:** Placement of IMUs according to Hindle et al. [1].

	Placement	Joint
**Foot**	*Distal to the lateral malleoli along the long axis of the foot.*	Ankle
**Shank**	*A 100 mm distal to the lateral femoral epicondyles along the long axis of the shank.*	
		Knee
**Thigh**	*A 150 mm proximal to the lateral femoral epicondyles along the long axis of the shank.*	
		Hip
**Pelvis**	*Midpoint between the posterior superior iliac spines, orthogonal to the ground.*	

**Table 2:** Mean ± SD (degrees) of joint angles and range of motion (ROM), *p*-value, and root mean square error (RMSE) during flat, unloaded walking.

	OMC	IMU	*p*	RMSE
Ankle				
*PFLX*	−0.9° ± 3.7°	25.3° ± 20.3°	**<0.001**	32.0°
*DFLX*	−34.0° ± 5.4°	−59.3° ± 17.6°	**<0.001**	29.6°
*ROM*	33.1° ± 5.4°	84.6° ± 18.5°	**<0.001**	52.5°
Knee				
*EXT*	−2.5° ± 5.7°	−16.6° ± 10.7°	**<0.001**	15.8°
*FLX*	66.5° ± 4.5°	46.8° ± 11.6°	**<0.001**	24.6°
*ROM*	69.0° ± 5.1°	63.4° ± 13.4°	0.118	18.5°
Hip				
*FLX*	34.9° ± 9.1°	18.7° ± 9.1°	**<0.001**	21.8°
*EXT*	−6.5° ± 9.1°	−23.7° ± 8.4°	**<0.001**	25.9°
*ROM*	41.4° ± 5.7°	42.4° ± 7.8°	0.679	12.6°

**Bold** is indicative of significant differences between OMC and IMU systems in plantarflexion (*PFLX*), dorsiflexion (*DFLX*), flexion (*FLX*), and extension (*EXT*).

**Table 3:** Mean ± SD (degrees) of joint angles and range of motion (ROM), *p*-value, and root mean square error (RMSE) during flat, unloaded running.

	OMC	IMU	*p*	RMSE
Ankle				
*PFLX*	11.8° ± 4.4°	44.0°± 21.8°	**<0.001**	38.3°
*DFLX*	−40.0° ± 8.0°	−79.9° ± 18.2°	**<0.001**	45.2°
*ROM*	51.8° ± 8.5°	123.9° ± 33.6°	**<0.001**	79.9°
Knee				
*EXT*	7.5° ± 5.2°	−40.9° ± 31.1°	**<0.001**	55.6°
*FLX*	84.0° ± 10.0°	73.0° ± 19.6°	**0.040**	21.9°
*ROM*	76.5° ± 11.6°	113.9° ± 48.2°	**0.004**	56.9°
Hip				
*FLX*	42.2° ± 9.8°	40.1° ± 23.1°	0.784	28.3°
*EXT*	−1.7° ± 6.2°	−56.3° ± 22.9°	**<0.001**	57.7°
*ROM*	43.9° ± 7.1°	96.4° ± 42.4°	**<0.001**	67.1°

**Bold** is indicative of significant differences between OMC and IMU systems in plantarflexion (*PFLX*), dorsiflexion (*DFLX*), flexion (*FLX*), and extension (*EXT*).

## Data Availability

The raw data supporting the conclusions of this article can be made available by the authors upon valid request to the corresponding author.

## Supplementary Materials

The following supporting information can be downloaded at https://www.mdpi.com/article/10.3390/s24051675/s1.

## Appendix A

Those who would like to use the MatLab application used for this study can download a copy of the application files and associated user help files (https://github.com/ALCFain/IMU_Application (accessed on 4 January 2024)). Note: This requires the aerospace toolbox of MatLab to run successfully.
