# Supplementary material for: IMUs Can Estimate Hip and Knee Range of Motion during Walking Tasks but Are Not Sensitive to Changes in Load or Grade
Source: Sensors (Basel). 2024 Mar 5;24(5):1675. doi: 10.3390/s24051675 (PMC10934173; doi:10.3390/s24051675)
Supplement: Supplementary file 1 [file sensors-24-01675-s001.zip › SupplementalTables(Sensitivity).pdf]

Table S1a: Mean ± SD (degrees) of joint angles and range of motion (ROM) for IMUs for each load (*unloaded, loaded*) and grade (*flat, uphill, downhill*) while walking.

	**Walking**					
	**Flat**		**Uphill**		**Downhill**	
**Ankle**	*Unloaded*	*Loaded*	*Unloaded*	*Loaded*	*Unloaded*	*Loaded*
PFLX	24.1° ± 18.9°	23.1° ± 11.8°	27.9° ± 18.4°	31.8° ± 34.1°	32.1° ± 32.7°	35.9° ± 23.9°
DFLX[b]	-57.9° ± 19.4°	-60.4° ± 15.9°	-62.2° ± 18.4°	-64.4° ± 15.2°	-68.3° ± 15.9°	-69.0° ± 20.5°
ROM[b]	81.9° ± 20.0°	83.5° ± 20.3°	90.1° ± 23.5°	96.2° ± 31.8°	100.4° ± 30.2°	104.9° ± 23.9°
**Knee**						
EXT	45.7° ± 11.3°	45.1° ± 13.6°	45.0° ± 9.7°	46.3° ± 12.9°	45.2° ± 16.0°	56.3° ± 19.6°
FLX[b]	-16.2° ± 11.1°	-21.9° ± 14.8°	-14.6° ± 14.2°	-15.0° ± 14.0°	-25.4° ± 20.8°	-30.5° ± 22.5°
ROM[a,b]	61.9° ± 14.7°	67.0° ± 11.6°	59.6° ± 11.4°	61.3° ± 13.8°	70.7° ± 23.7°	86.8° ± 32.7°
**Hip**						
FLX	17.7° ± 9.7°	23.1° ± 10.7°	19.1° ± 17.6°	23.6° ± 28.4°	21.8° ± 10.8°	24.4° ± 14.6°
EXT	-25.9° ± 14.0°	-24.1° ± 11.2°	-20.6° ± 21.1°	-19.5° ± 30.1°	-23.2° ± 7.2°	-23.7° ± 12.9°
ROM	43.6° ± 10.7°	47.2° ± 11.2°	39.7° ± 7.7°	43.1° ± 10.2°	45.1° ± 10.3°	48.0° ± 15.7°

Table S1b: Mean ± SD (degrees) of joint angles and range of motion (ROM) for IMUs for each load (*unloaded, loaded*) and grade (*flat, uphill, downhill*) while running.

	**Running**					
	**Flat**		**Uphill**		**Downhill**	
**Ankle**	*Unloaded*	*Loaded*	*Unloaded*	*Loaded*	*Unloaded*	*Loaded*
PFLX	44.3° ± 21.5°	38.6° ± 17.3°	45.1° ± 25.7°	40.8° ± 21.5°	42.9° ± 21.3°	38.5° ± 23.2°
DFLX	-79.7° ± 18.4°	-72.6° ± 18.7°	-75.5° ± 23.3°	-80.0° ± 18.9°	-77.5° ± 23.9°	-80.7° ± 21.5°
ROM	124.0° ± 31.9°	111.2° ± 26.2°	120.6° ± 43.2°	120.8° ± 29.5°	120.4° ± 29.8°	119.2° ± 33.0°
**Knee**						
EXT[b]	76.7° ± 22.3°	72.7° ± 24.9°	82.0° ± 26.5°	77.3° ± 21.7°	72.8° ± 24.5°	71.5° ± 21.2°
FLX	-44.6° ± 35.5°	-38.0° ± 35.7°	-46.7° ± 41.6°	-51.8° ± 42.4°	-44.7° ± 30.5°	-42.4° ± 31.2°
ROM	121.4° ± 55.0°	110.6° ± 58.1°	128.7° ± 66.3°	129.1° ± 61.2°	117.5° ± 51.2°	113.9° ± 49.0°
**Hip**						
FLX	42.9° ± 27.7°	34.8° ± 25.6°	40.3° ± 25.5°	42.0° ± 25.1°	44.2° ± 29.9°	39.0° ± 27.6°
EXT[a]	-56.3° ± 21.5°	-46.0° ± 22.2°	-59.9° ± 20.5°	-43.1° ± 23.1°	-52.7° ± 26.8°	-44.5° ± 16.8°
ROM[a]	99.2° ± 43.0°	80.7° ± 41.3°	100.2° ± 42.2°	85.2° ± 40.3°	96.8° ± 49.3°	83.4° ± 35.8°

Effect of load[a], Effect of grade[b], Effect of load and grade[c] on plantarflexion (PFLX), dorsiflexion (DFLX), flexion (FLX), and extension (EXT).

Table S2a: Mean ± SD (degrees) of joint angles and range of motion (ROM) for OMC for each load (unloaded, loaded) and grade (flat, uphill, downhill) while walking.

	**Walking**					
	**Flat**		**Uphill**		**Downhill**	
**Ankle**	*Unloaded*	*Loaded*	*Unloaded*	*Loaded*	*Unloaded*	*Loaded*
PFLX[b]	-0.3° ± 3.7°	12.4° ± 1.4°	-0.0° ± 4.1°	12.2° ± 3.8°	2.0° ± 3.6°	16.1° ± 5.1°
DFLX[b]	-33.8° ± 5.5°	-38.6° ± 8.7°	-34.3° ± 5.5°	-38.4° ± 7.8°	-35.9° ± 6.7°	-39.4° ± 8.5°
ROM[b]	33.4° ± 5.5°	50.9° ± 8.73°	34.3° ± 5.5°	50.6° ± 7.4°	37.9° ± 6.7°	55.5° ± 9.1°
**Knee**						
EXT[a,b,c]	-3.2° ± 5.7°	8.0° ± 5.2°	1.1° ± 6.0°	12.2° ± 7.3°	1.6° ± 6.1°	10.3° ± 6.2°
FLX[a,b,]	67.1 ± 4.6°	84.9° ± 10.1°	68.7° ± 5.1°	85.2° ± 9..4°	65.7° ± 4.6°	90.4° ± 10.4°
ROM[a,c]	70.3° ± 5.9°	76.9° ± 11.1°	68.6° ± 5.5°	73.9° ± 10.4°	65.1° ± 5.0°	80.1° ± 13.5°
**Hip**						
FLX[a,b]	35.3° ± 8.0°	43.3° ± 9.5°	38.0° ± 8.5°	49.1° ± 10.9°	41.3° ± 8.3°	50.0° ± 10.1°
EXT	-6.0° ± 8.7°	-1.5° ± 6.0°	-5.5° ± 7.7°	-0.3° ± 6.5°	-6.2° ± 8.3°	-1.5° ± 6.5°
ROM[a,b,c]	41.3° ± 5.3°	44.9° ± 6.6°	43.5° ± 4.6°	49.4° ± 7.7°	47.5° ± 5.2°	51.5° ± 7.4°

Table S2b: Mean ± SD (degress) of joint angles and range of motion (ROM) for OMC for each load (unloaded, loaded) and grade (flat, uphill, downhill) while running.

	**Running**					
	**Flat**		**Uphill**		**Downhill**	
**Ankle**	*Unloaded*	*Loaded*	*Unloaded*	*Loaded*	*Unloaded*	*Loaded*
PFLX[b]	2.5° ± 3.9°	16.4° ± 3.4°	0.3° ± 4.3°	8.5° ± 3.6°	1.4° ± 5.1°	8.3° ± 4.2°
DFLX[b]	-35.1° ± 5.7°	-39.1° ± 8.2°	-30.0° ± 5.9°	-35.4° ± 9.8°	-29.8° ± 5.7°	-35.3° ± 8.3°
ROM[b]	37.6° ± 5.2°	55.5° ± 8.0°	30.0° ± 5.5°	43.9° ± 10.0°	31.2° ± 5.1°	43.6° ± 7.9°
**Knee**						
EXT[a,b]	3.1° ± 6.3°	14.3° ± 7.6°	-3.9° ± 5.7°	3.9° ± 5.7°	-0.6° ± 6.0°	6.3° ± 6.9°
FLX[b]	68.3° ± 5.0°	88.1° ± 9.8°	70.6° ± 4.3°	83.5° ± 10.4°	73.6° ± 5.0°	84.1° ± 11.8°
ROM[a,b,c]	66.2° ± 5.8°	73.7° ± 13.7°	73.7° ± 6.0°	79.6° ± 10.8°	74.1° ± 6.1°	77.8° ± 22.4°
**Hip**						
FLX[a,b,c]	45.5° ± 9.4°	56.1° ± 11.1°	32.6° ± 8.1°	39.5° ± 9.4°	35.7° ± 8.4°	43.8° ± 11.2°
EXT[a]	-6.0° ± 7.4°	-0.8° ± 7.0°	-5.5° ± 8.1°	-0.9° ± 6.7°	-4.4° ± 8.1°	0.9° ± 6.4°
ROM[a,b,c]	51.5° ± 5.7°	56.9° ± 8.6°	38.0° ± 3.6°	40.4° ± 6.3°	40.1° ± 3.9°	42.9° ± 7.4°

Effect of load[a], Effect of grade[b], Effect of load and grade[c] on plantarflexion (PFLX), dorsiflexion (DFLX), flexion (FLX), and extension (EXT).
